# The Possible Role of *Helicobacter pylori* Infection in Non-alcoholic Fatty Liver Disease

**DOI:** 10.3389/fmicb.2017.00743

**Published:** 2017-05-10

**Authors:** Dan-dan Cheng, Cong He, Hong-hui Ai, Ying Huang, Nong-hua Lu

**Affiliations:** ^1^Department of Gastroenterology, The First Affiliated Hospital of Nanchang UniversityNanchang, China; ^2^Department of Orthopaedics, The Yugan County People's HospitalYugan, China

**Keywords:** *H. pylori*, NAFLD, insulin resistance, lipid profile, gut microbiota

## Abstract

*Helicobacter pylori* (*H. pylori*) which colonizes the stomach can cause a wide array of gastric disorders, including chronic gastritis, peptic ulcer, and gastric cancer. Recently, accumulating evidence has implicated *H. pylori* infection in extragastrointestinal diseases such as cardiovascular diseases, neurological disorders, and metabolic diseases. At the same time, many scholars have noted the relationship between *H. pylori* infection and non-alcoholic fatty liver disease (NAFLD). Despite the positive association between *H. pylori* and NAFLD reported in some researches, there are opposite perspectives denying their relationship. Due to high prevalence, unclear etiology and difficult treatment of NAFLD, confirming the pathogenicity of *H*. p*ylori* infection in NAFLD will undoubtedly provide insights for novel treatment strategies for NAFLD. This paper will review the relationship between *H. pylori* infection and NAFLD and the possible pathogenic mechanisms.

## Introduction

*Helicobacter pylori* (*H. pylori*) is a gram-negative microaerophilic bacterium that colonizes the stomach of humans. In 1983, Australian researchers Marshall and Warren (1983) first successfully isolated and cultured *H. pylori* from the human body. So far, *H. pylori* is one of the most common human infectious bacteria with a worldwide prevalence of ~50%. In the United States and Europe, the prevalence of *H. pylori* is estimated to be 20–50%, varying in different socioeconomic, age and ethnic groups, and geography (Ford and Axon, 2010). In developing countries, the prevalence has been reported to be as high as 70% (Mandeville et al., 2009). We all know that *H. pylori* infection is one of the most important environmental risk factors for the diseases of chronic gastritis, peptic ulcers, gastric mucosa-associated lymphoid tissue (MALT) lymphoma, and gastric cancer. Recently, accumulating evidence implicates *H. pylori* infection existing in the patients with extragastrointestinal diseases, including idiopathic thrombocytopenic purpura, ischemic heart diseases, obesity, type 2 diabetes mellitus (T2DM) (Wong et al., 2014; Dogan et al., 2015; Nasif et al., 2016). In addition, there is a special focus on the relationship between *H. pylori* infection and NAFLD.

Unlike alcoholic fatty liver disease, the patients with NAFLD should be an alcohol consumption of < 10 g per day (Neuschwander-Tetri and Caldwell, 2003). NAFLD comprises a spectrum of diseases ranging from simple non-alcoholic fatty liver (NAFL) and non-alcoholic steatohepatitis (NASH) to fibrosis, liver cirrhosis, and ultimately hepatocellular carcinoma (HCC) (Caldwell and Argo, 2010). NAFL is thought to be a relatively benign state, while NASH represents a form of NAFLD that can potentially progress to cirrhosis and HCC. Due to changes in dietary habits and an increase in the number of people engaged in a sedentary lifestyle, the prevalence of NAFLD is increasing worldwide over time (Bhala et al., 2013), which may seriously influence human's health and the quality of life. The worldwide prevalence of NAFLD in the general population is estimated to be 20–30% in Western countries and 5–18% in Asia and is increasing over time (Masarone et al., 2014). Up to 25% of NAFLD patients will evolve into a progressive form of liver diseases named non-alcoholic steatohepatitis (NASH), which is currently the second leading etiology of liver diseases among the adults awaiting liver transplantation in the United States. To date, the exactly pathogenesis mechanisms of NAFLD remain unknown. We know that *H. pylori* infection has been implicated in the pathogenesis of insulin resistance (IR) (Polyzos et al., 2011), which contributes to the development of NAFLD. Recently, many studies have reported that *H. pylori* infection is closely related to the development of NAFLD (Polyzos et al., 2013a; Sumida et al., 2015). However, the mechanisms underlying NAFLD remains unclear, and therapeutic options to this disorder are fairly limited nowadays. Thus, investigating the role of *H. pylori* infection as a risk factor for IR might facilitate understanding its effects on NAFLD. The identification of novel targets for NAFLD therapy is of high priority. Treatment for *H. pylori* infection is easy and relatively inexpensive, and the interest in exploring its involvement in arising extra-gastric manifestations, is of great interest for public health.

## Epidemiology and clinical experiments

Specific studies regarding the association between *H. pylori* infection and NAFLD are increasing. Firstly, *H. pylori* deoxyribonucleic acid (DNA) has been detected in patients with various etiologies of chronic liver diseases (CLD), including hepatitis, liver fibrosis, and HCC (De Magalhaes Queiroz and Santos, 2001; Castera et al., 2006; Pellicano et al., 2008). In 2008, Cindoruk et al. first found the presence of 16S recombinant RNA of *H. pylori* in the liver sample of a 44-year-old patient with NASH (Cindoruk et al., 2008). This finding was then been validated by another study, in which the *H. pylori* DNA was found in 5/11 liver samples of NAFLD patients compared with 2/13 controls (Pirouz et al., 2009). In 2009, it has been demonstrated in an animal model of *H. pylori* infection that *H. pylori* inoculated orally, could arrive in the liver and cause hepatitis, further suggested a causative role of *H. pylori* in CLD (Huang et al., 2009). Surprisingly, *H. pylori* sequence were found in the liver tissues of chronic hepatitis C patients even though the serology of *H. pylori* was negative (Castera et al., 2006). The authors speculated two possible mechanisms for the presence of *H. pylori* in the liver: the bacterium may pass from the stomach to the liver through the duodenum and biliary tract, or may arrive in the liver from the circulation through the hepatic portal vein (Pellicano et al., 2008). Some data indicate the biliary pathway as the most plausible route (Tiwari et al., 2006; Aviles-Jimenez et al., 2016).

Since then, a train of clinical studies of the relationship between *H. pylori* infection and NAFLD were successively reported (Table 1; Takuma, 2011; Kountouras et al., 2014; Zhang et al., 2016). At the earliest, a study from Greece recruiting 28 patients with biopsy-confirmed NAFLD (15 with NAFL, and 13 with NASH) and 25 matched healthy controls found higher rates of anti-*H. pylori* IgG in NAFLD groups compared to control group (*p* = 0.038) and *H. pylori* infection could independently predict NAFLD in logistic regression analysis. It is indicated that *H. pylori* infection may represent one more hit contributing to the pathogenesis of NAFLD (Polyzos et al., 2013a). In 2015, another Japanese clinical study involving 130 patients with biopsy-confirmed NAFLD (43 with NAFL and 87 with NASH) found that the prevalence of NASH was significantly higher in patients with *H. pylori* IgG seropositivity than in those without (81 and 58%, *p* = 0.008). Besides, the total NAFLD activity score (NAS) and the grade of hepatocyte ballooning were higher in patients with *H. pylori* IgG seropositivity than in those without (*p* = 0.03). This study also found that *H. pylori* infection could independently predict NASH in logistic regression analysis (*p* = 0.003). And it further confirmed that *H. pylori* infection may represent a contributing factor to NAFLD (Sumida et al., 2015). Similarly, another two studies from Turkey and Japan, respectively, also suggested *H. pylori* infection as one of the independent risk factors for the development of NAFLD (Takuma, 2011; Sumida et al., 2015). Moreover, Abenavoli et al. (2013) described a case report in which the metabolic profile of a 55-year old man, including the homeostatic model assessment of insulin resistance (HOMA-IR), fatty liver index and echographic liver pattern, was improved after *H. pylori* eradication. It further supports the significance of *H. pylori* infection in the development of NAFLD. However, there were some studies declared that *H. pylori* infection is not related to NAFLD (Polyzos et al., 2014; Okushin et al., 2015; Baeg et al., 2016). In recent years, two studies from Korea and Japan involved 3,663 and 13,737 patients, respectively, found that *H. pylori* infection was not associated with NAFLD and may not be the risk factor for NAFLD (Okushin et al., 2015; Baeg et al., 2016). But, one of the drawbacks of these two studies is the limitation of liver ultrasonography for diagnosis and grading the severity of NAFLD. So, taking into account the differences in diagnosis and the small sample size, clinical trials with larger sample sizes are needed to confirm the exact relationship between *H. pylori* and NAFLD. If this correlation is verified, the treatment of *H. pylori* may represent a new specific therapeutic strategy for NAFLD.

**Table 1:** **Overview of studies regarding the effect of ***H. pylori*** infection and NAFLD**.

**References**	**Country**	**Journal**	**Year**	**Study design**	**Sample population**	**Test for *H. pylori* infection**	**Diagnostic criteria for NAFLD**	**Results**
Polyzos et al., 2013a	Greece	Metabolism	2013	Single-center, cross-sectional study	NAFLD: *n* = 28	Serum anti-*H. pylori* UBT	Liver biopsy	Higher rates of anti- *H. pylori* IgG were observed in NAFLD compared to control group. Both *H. pylori* infection and log(HOMA-IR) could independently predict NAFLD in logistic regression analysis.
					Controls: *n* = 25			
Sumida et al., 2015	Japan	J. Gastroenterol.	2015	Single-center, cross-sectional study	*n* = 130	Serum anti-*H. pylori*	Liver biopsy	The prevalence of NASH was significantly higher in the patients with *H. pylori* IgG seropositivity than in those without.
Dogan et al., 2013	Japan	Eur. J. Gastroenterol. Hepatol.	2013	Randomized-controlled single-blind study	*H. pylori* (+): *n* = 95	UBT	Liver ultrasonography	Fatty liver was found significantly more frequently in the *H. pylori-*positive group. A relationship between *H. pylori* and fatty liver was observed with univariate analysis.
					*H. pylori* (−): *n* = 79			
Abenavoli et al., 2013	Italy	Med. Hypotheses	2013	Case report	A 55-year man	UBT	Liver ultrasonography	He is improved the metabolic profile including insulin resistance, fatty liver index and echographic liver after the treatment for *H. pylori* eradication.
Baeg et al., 2016	Korea	WJG	2016	Single-center, cross-sectional study	*H. pylori* (+): *n* = 1636	UBT	NAFLD-LFS = −2.89 + 1.18 × metabolic syndrome (yes = 1, no = 0) + 0.45 × type 2 diabetes (yes = 2, no = 0) + 0.15 × insulin (mU/L) + 0.04 × AST (U/L) −0.94 × AST/ALT	*H. pylori* infection is not a risk factor for NAFLD as indicated by HSI or NAFLD-LFS.
					*H. pylori* (−): *n* = 2027			
Jamali et al., 2013	Iran	Hepat. Mon.	2013	Randomized open-label clinical trial	*N* = 100	UBT	Liver ultrasonography	It seems that *H. pylori* eradication *per se* might not affect LFC, LFT, lipid profile, and insulin resistance in dyspeptic NAFLD patients.
Okushin et al., 2015	Japan	BMC Gastroenterol.	2015	Single-center, cross-sectional study	FLD: *n* = 6404	Serum anti-*H. pylori*	Liver ultrasonography	Body mass index, serum ALT and platelet count were significantly associated with FLD and NAFLD, whereas infection of *H. pylori* was not.
					Controls: *n* = 7333			

## The possible mechanisms of the influence of *H. pylori* infection on NAFLD

### Insulin resistance (IR)

IR is closely associated with NAFLD and is also one of the independently risk factors of it (Wang et al., 2013; Birkenfeld and Shulman, 2014). Early in 1998, Day and James (1998) firstly proposed the “two hits” theory for the pathogenesis of NAFLD. They put forward that, the first strike mainly referred to the excessive accumulation of fat in the liver parenchymal cells. This process is associated with IR, which can lead to a dysfunction of intracellular triglyceride synthesis and transport. Recently, some researchers proposed the “multiple hits” hypothesis, which is now widely accepted. They considered multiple insults that interact together on genetically predisposed subjects and induce NAFLD, which provides a more accurate explanation of NAFLD pathogenesis. Such hits include insulin resistance, hormones secreted from the adipose tissue, nutritional factors, gut microbiota, and genetic and epigenetic factors (Takaki et al., 2013; Buzzetti et al., 2016).

In 2005, Aydemir et al. first directly confirmed the association between chronic *H. pylori* infection and IR, and their results showed that the HOMA-IR of subjects in the *H. pylori* positive group was significantly higher than that in the *H. pylori* negative group (*p* < 0.05; Aydemir et al., 2005). In 2009, a large cross-sectional study including 1,107 subjects, found that the *H. pylori* seropositivity rate was significantly higher for patients in the IR group (HOMA-IR ≥ 2.5) than in patients in the non-IR group (HOMA-IR < 2.5; 39.4 vs. 28.7%, respectively, *p* = 0.027). Multiple linear regression analysis showed that *H. pylori* infection was significantly correlated with the HOMA-IR (95% CI = 0.058–0.246, *p* = 0.001). The authors therefore proposed that *H. pylori* infection may be an important independent risk factor for the development of IR (Gunji et al., 2009). Recently, a study of *H. pylori* infection in NAFLD showed that the rate of infection in NAFLD group is higher than control group, and both *H. pylori* infection (*p* = 0.034) and log (HOMA-IR) (*p* = 0.007) could independently predict NAFLD. Moreover, the study also examined the levels of glucose, insulin, HOMA-IR, ALT, AST, and TNF-α, and found that all of these items were higher in *H. pylori*-IgG positive group compared to negative group. Therefore, the author put forward that *H. pylori* infection may contribute to the pathogenesis of NAFLD, mainly by adding to the first hit and this process may be achieved indirectly, or directly, though increasing IR (Polyzos et al., 2013a). Many other studies also demonstrated the causal relationship between *H. pylori* and IR (Polyzos et al., 2013b; Chen et al., 2015). A prospective study reported that after *H. pylori* eradication, the fasting plasma insulin level (*p* < 0.01) and HOMA-IR (*p* < 0.01) were significantly lower than before treatment, indicating that *H. pylori* eradication improved IR and may prevent the occurrence of MS, and NAFLD (Abenavoli et al., 2013; Dogan et al., 2015).

The *H. pylori*-induced IR in NAFLD may be indirectly by causing chronic inflammation or directly by activating certain signaling pathway. Many basic and clinical studies have confirmed that chronic inflammation plays an important role in IR (Hossain et al., 2016). Studies have reported that chronic inflammation due to *H. pylori* infection can increase the expression of C-reactive protein (CRP), tumor necrosis factor (TNF)-α, and interleukin (IL)-6 (Tsai et al., 2015; Yildirim et al., 2016). These inflammatory cytokines would activate a series of kinases such as IKK/NF-kB and JNK, eventually trigger IR by up-regulating Ser-phosphorylation (Hotamisligil et al., 1996) or inhibition of the autophosphorylation of the tyrosyl of the insulin receptor substrate (IRS)-1 (Dandona et al., 2004). Another study through establishing two kinds of mice models of *H. pylori* infection and high-fat-diat-fed group, detecting insulin signaling pathway, and relative protein and RNA level in liver tissue, demonstrated that *H. pylori* infection inhibited miR-203 expression through c-Jun overexpression, and then resulting in the induction of SOCS3, which is a well-known inhibitor of insulin signaling. It manifested *H. pylori* infection caused hepatic IR by the c-Jun/miR-203/SOCS3 signaling pathway (Zhou et al., 2015).

## Inflammation cytokines or adipocytokines

The pathogenesis of NASH was originally conceptualized as a disease of consecutive hits: the accumulation of fat in the liver cells (steatosis) that sensitized the liver to a second metabolic insult triggering a cascade of tissue damage (inflammation) resulting in fibrosis. Although, the etiology of NAFLD is maltifactorial and remains largely enigmatic, it is well-accepted that inflammation is a central component of NAFLD pathogenesis (Peverill et al., 2014). A variety of inflammatory cytokines are involved in *H. pylori* infection, with the closest relationships detected among CRP, TNF-α, IL-6, and interleukin (IL)-1β (Shoelson et al., 2006; Keane et al., 2015). The levels of CRP, TNF-α, and IL-6 in the serum reflect low-grade chronic inflammation in human (Silha et al., 2007). A study regarding *H. pylori* infection in NAFLD, found TNF-α and IR were significant higher and circulating adiponectin is lower in *H. pylori* seropositivity compared to *H. pylori* seronegativity. *H. pylori* infection may trigger TNF-α, whereas adiponection is secondarily increased to counterbalance the pro-inflammatory cascade. This may be achieved indirectly, through increasing IR which is descripted above, but also directly, given that it could predict NAFLD independently from IR. TNF-α may be a mediator of both direct and indirect effect of *H. pylori* infection on NAFLD (Polyzos et al., 2013a). CRP is synthesized in liver and adipocytes upon IL-6 and TNF-α stimulation. Hs-CRP levels showed higher in NASH cases vs. non-NASH cases, and it can be used as a non-invasive biomarkers of NAFLD (Maleki et al., 2014). Polyzos's study also detected hs-CRP in *H. pylori* infection of NAFLD patients, the results showed that the hs-CRP level was higher in *H. pylori* positive group than in *H. pylori* negative group (Polyzos et al., 2013a). A prospective, open-label, single-center study involving 159 *H. pylori* positive patients, who received a 14-day sequential regimen, the results showed that the HOMA-IR and CRP level were significantly higher in patients with *H. pylori* infection compared to the patients without *H. pylori* infection (*P* < 0.05). While, 6 weeks after eradication therapy, the two indicators were significantly decreased from the pretreatment level (*P* < 0.05; Gen et al., 2010). Thus, we speculate that the *H. pylori* related-inflammation may play a role in the pathogenesis of NAFLD. The mechanisms of the pathogenesis of *H. pylori*-related inflammation in NAFLD are directly reducing hepatocyte glycogen levels via a JNK signaling pathway (Li et al., 2010), which can downregulate the expression of key genes of glucose and accelerate lipolysis (Hotamisligil et al., 1996), and indirectly inducing IR through some pathways, which we list above.

In addition, adipose tissue is not only involved in energy metabolism but also contributes to IR by secreting cytokines such as leptin and adiponectin and participates in the development of NAFLD. Studies suggested that the levels of plasma leptin were elevated in NAFLD group (Friedman, 2008), and it is an independent predictor of hepatic steatosis (Chitturi et al., 2002; Polyzos et al., 2016). A study involving 153 dyspeptic patients, found that *H. pylori* infection was significantly negatively associated with serum leptin level (*P* < 0.001). *H. pylori* infection may influence leptin production (Hemmasi et al., 2013), which can inhibit liver stearoyl-CoA desaturase, thus reducing VLDL-C and fatty deposits in the liver tissue (Ding et al., 2005). Besides, leptin may phosphorylate IRS serine 1,318, thus interfering with insulin signal transduction (Hennige et al., 2006). And it is considered to be a pro-inflammatory cytokine and has structural similarity to other pro-inflammatory cytokines such as IL-6 and IL-12 (La Cava and Matarese, 2004). So we rationally speculate that *H. pylori* infection may cause NAFLD by influencing fat metabolism and transporting relevant enzymes or by insulin signal transduction. But, other studies revealed that *H. pylori* infection accelerated the synthesis of leptin (Nishi et al., 2005). Thus, more investigation is needed to determine whether there are relationships between *H. pylori* and serum leptin levels which could account for the discrepancies.

### Lipid profiles

A standard histological feature of NAFLD is the presence of at least 5% hepatocyte steatosis (Tiniakos, 2010). A common feature of NAFLD is the presence of the hepatic ectopic fat deposition (Liu et al., 2010). A study on the association between hepatic lipids and insulin clearance showed that liver lipid content was significantly linked to insulin clearance (*r* = 0.43, *p* < 0.0001) and hepatic insulin sensitivity (*r* = −0.04, *p* = 0.0002; Kotronen et al., 2007, 2008). Therefore, IR contributes to liver lipid deposition, while increased hepatic lipid accumulation in turn further aggravates IR, leading to a vicious cycle and promoting the development of liver steatosis and NAFLD. This cycle increases the triglycerides (TG) and free fatty acid (FFA) content in liver cells, producing a serious burden for the metabolism of lipids in hepatocytes. Lipid metabolism further produces large amounts of superoxide anions and reactive oxygen species, leading to liver fat peroxidation and oxidative stress (Vergun and Reynolds, 2005). Thus, this kind of vicious cycle will give a heavy blow to hepatocyte.

In 1999, Laurila et al. found that the serum TG and total cholesterol (T-CHOL) concentrations were significantly higher in the males with positive IgG antibody titers for *H. pylori* than in the males with no signs of infection (*p* < 0.001), indicating that chronic *H. pylori* infection may influence the serum lipid profile (Laurila et al., 1999). Recently, a meta-analysis on the association between *H. pylori* infection and metabolic syndrome involving 27,544 participants showed that, compared to *H. pylori* negative groups, *H. pylori* positive groups were lower in HDL-C (MD = −2.43, 95% CI: −3.75 to −1.12, *I*^2^ = 92%) and higher in TG (MD = 8.12, 95% CI: 3.05–13.20, *I*^2^ = 71%; Upala et al., 2016). Nam et al. (2015) involving 13,383 participants also found that current infection with *H. pylori* with 50.5% at baseline increased LDL-C and decreased HDL-C than *H. pylori* negative group. A study involving in 679 participants, with UPLC-MS analysing the concentrations of molecular lipids, and proton magnetic resonance spectroscopy (^1^H-MRS) or liver biopsy measuring liver-fat, demonstrated that a serum-lipid signature comprising a lipid triplet (TG[16:0/18:0/18:1], phosphatidylcholine [PC][18:1/22:6], PC[O-24:1/20:4]) could estimate the percentage of liver fat. The significant associations of specific lipid and polar metabolite concentrations with liver-fat content suggested that circulating molecular lipids may be predictive of liver fat (Oresic et al., 2013). Hyperlipidemia is an established risk factor of NAFLD. A prospective, open-label, single-center study which consisted of 159 NAFLD patients, revealed that the HOMA-IR, TC, TG, LDL-C, and CRP levels were significantly higher and HDL-C levels were significantly lower in patients with *H. pylori* infection (*P* < 0.05), while HOMA-IR, TC, TG, LDL-C, and CRP levels in patients with successful eradication were significantly decreased compared to the pretreatment levels (*P* < 0.05). This study showed beneficial effects of *H. pylori* eradication on insulin resistance, atherogenic lipid abnormalities, and low-grade inflammation. These results suggest that *H. pylori* eradication may prevent metabolic syndrome including NAFLD (Gen et al., 2010). However, the mechanism by which this occurs is not yet clear.

### Intestinal permeability and gut microbiota

In recent years, as the maturity and widely applying of 16s rRNA gene sequencing technique, evidence linking dysbiosis to the pathogenesis of human liver disease has accumulated rapidly, with a primary focus on its role in NAFLD. Animal studies in which the gut microbiota are manipulated, and observational studies in patients with NAFLD, have provided considerable evidence that gut microbiota dysbiosis contributes to the pathogenesis of NAFLD (Turnbaugh et al., 2006; Le Roy et al., 2013; Mouzaki et al., 2013; Jiang et al., 2015; Leung et al., 2016). A retrospective study described a significant imbalance in intestinal flora in patients with NAFLD and intestinal inflammation. NAFLD significantly increased the permeability of the intestinal mucosa, and the author postulated that aside from dysbiosis of the gut microbiota, gut microbiota-mediated inflammation of the intestinal mucosa and the related impairment in mucosal immune function play an important role in the pathogenesis of NAFLD (Jiang et al., 2015). Another retrospective study from France enrolled 57 patients with biopsy-proven NAFLD also revealed that, Bacteroides abundance was significantly increased in NASH and *F* ≥ 2 patients, whereas Prevotella abundance was decreased, and Ruminococcus abundance was significantly higher in *F* ≥ 2 patients. By multivariate analysis, Bacteroides abundance was independently associated with NASH and Ruminococcus with *F* ≥ 2 fibrosis (Boursier et al., 2016). Dysbiosis increases gut permeability to bacterial products and increases hepatic exposure to injurious substances that increase hepatic inflammation and fibrosis.

Fukuda et al. (2001) evaluated the effect of *H. pylori* infection on the permeability of the intestine by oral sucrose tolerance test. The results showed that the existence of *H. pylori* itself was associated with increased intestinal permeability. Myllyluoma et al. (2007) observed the difference with regard to clostridia and the total number of anaerobic bacteria by detecting the composition of the microbiota between *H. pylori*-positive and *H. pylori*-negative individuals. Heimesaat et al. (2014) demonstrated that long-term infection of Mongolian gerbils with an *H. pylori* WT strain leads to distinct shifts of the microbiota composition in the distal uninflamed intestine. Khosravi et al. (2015) observed that *H. pylori* infection was related to gut microbiota using germ free (GF) and specific pathogen free (SPF) mice in the presence and absence of *H. pylori*. The results also indicated that ongoing crosstalk occurs between *H. pylori* and the normal gut microbiota, which is associated with metabolism and gut inflammation. Thus, some scholars speculate the mechanisms of the development of NAFLD caused by *H. pylori* related-gut microbiota dysbiosis are follows; *H. pylori* invasion into intestinal mucosa might increase gut permeability and gut microbiota disorder and then facilitate the passage of bacterial endotoxin (mainly LPS) via the portal vein to the liver, and promote inflammation response (Fukuda et al., 2001; Sumida et al., 2015).

## Conclusion and outlook

**Figure 1:**
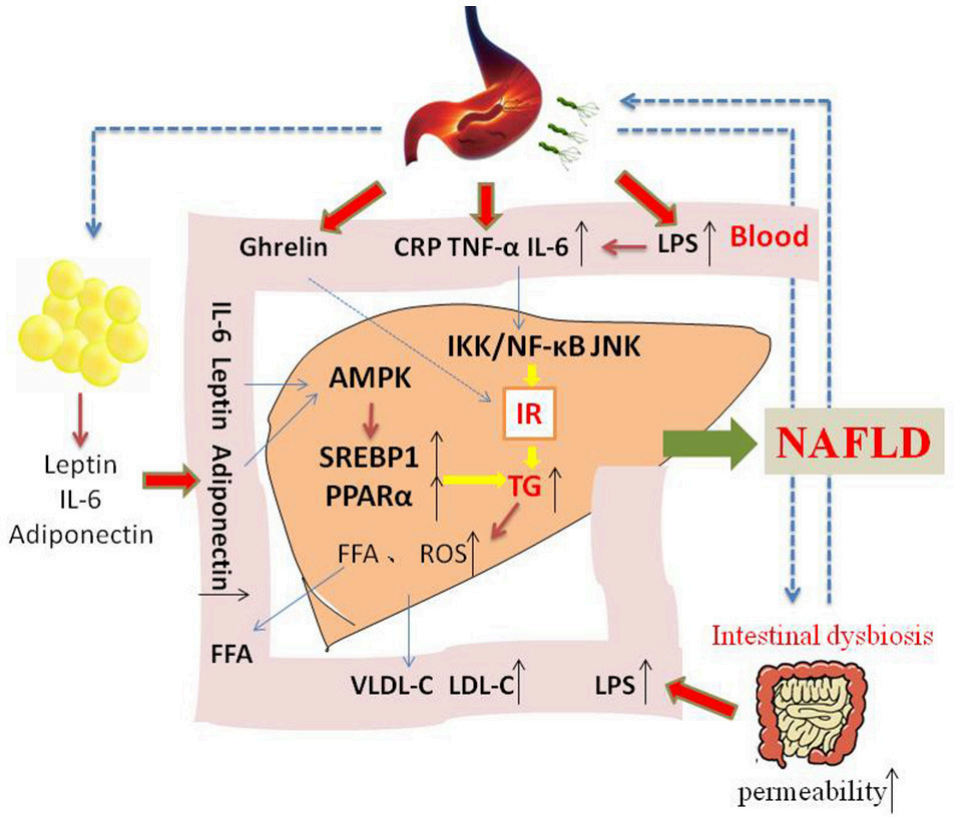
**The possible mechanism of how ***H. pylori*** infection contribute to NAFLD**. Insulin resistance is the central to the development of NAFLD. On the one hand, *H. pylori* infection may cause chronic low-grade systemic inflammation, increasing the levels of inflammatory cytokines such as IL-6 and TNF-α, which may influence insulin action and its level. On the other hand, *H. pylori* infection may also stimulate white adipose tissue to release leptin and adiponectin, activating AMPK and then upregulating SREBP1c and PPARα. In addition, other mechanism and mediators may be involved in the possible causative relationship between *H. pylori* infection and NAFLD.

NAFLD is a complex disease that is affected by genetic and environmental factors. The incident rate is high, and most of the patients have a good prognosis, however, up to 25% probability develop to NASH, cirrhosis, or liver cancer. Thus, effective treatment regimen for prevention of this progress are extremely urgent. IR is considered to be an crucial part of NAFLD development, and many studies have confirmed that *H. pylori* infection may be a causal factor for IR. The mechanisms of *H. pylori* infection that contribute to NAFLD may include the following. *H. pylori* infection may cause chronic low-grade systemic inflammation, increasing the levels of inflammatory cytokines such as IL-6 and TNF-α, then activating IKK/NF-KB and leading to IR. *H. pylori* infection may also inhibit white adipose tissue to release leptin, and then promote liver stearoyl-CoA desaturase, thus accelerating VLDL-C and fatty deposits in the liver tissue. Due to the interaction of the stomach and the intestines, *H. pylori* infection may lead to gastrointestinal flora dysbiota, and increasing serum LPS, stimulating systemic inflammation and causing a decrease in lipoprotein activity followed by dyslipidemia (Figure 1). If we can understanding and confirming the pathogenic role of *H. pylori* infection in NAFLD, it will provide a new direction for NAFLD treatment strategies.

## Author contributions

NL gives the direction of the paper's conception. DC writes the manuscript. CH helps to combing logic and HA, YH helps in modifying language.

## Funding

Supported by The National Natural Science Foundation of China (Nos. 81270479, 81470832 and 81670507).

### Conflict of interest statement

The authors declare that the research was conducted in the absence of any commercial or financial relationships that could be construed as a potential conflict of interest.
